# Use of machine learning to predict the risk of early morning intraocular pressure peaks in glaucoma patients and suspects

**DOI:** 10.5935/0004-2749.20210101

**Published:** 2025-08-21

**Authors:** Camilo Brandão-de-Resende, Sebastião Cronemberger, Artur W. Veloso, Rafael V. Mérula, Carolina S. Freitas, Érica A. Borges, Alberto Diniz-Filho

**Affiliations:** 1 Hospital São Geraldo, Universidade Federal de Minas Gerais, Belo Horizonte, MG, Brazil; 2 Department of Ophthalmology and Otorhinolaryngology, Universidade Federal de Minas Gerais, Belo Horizonte, MG, Brazil

**Keywords:** Glaucoma, Glaucoma, open-angle, Ocular hypertension, Intraocular pressure, Machine learning, Glaucoma, Glaucoma de ângulo aberto, Suspeita de glaucoma, Pressão intraocular, Aprendizado de máquina

## Abstract

**Purpose:**

To use machine learning to predict the risk of intraocular pressure peaks at 6 a.m. in primary open-angle glaucoma patients and suspects.

**Methods:**

This cross-sectional observational study included 98 eyes of 98 patients who underwent a 24-hour intraocular pressure curve (including the intraocular pressure measurements at 6 a.m.). The diurnal intraocular pressure curve was defined as a series of three measurements at 8 a.m., 9 a.m., and 11 a.m. from the 24-hour intraocular pressure curve. Two new variables were introduced: slope and concavity. The slope of the curve was calculated as the difference between intraocular pressure measurements at 9 a.m. and 8 a.m. and reflected the intraocular pressure change in the first hour. The concavity of the curve was calculated as the difference between the slopes at 9 a.m. and 8 a.m. and indicated if the curve was bent upward or downward. A classification tree was used to determine a multivariate algorithm from the measurements of the diurnal intraocular pressure curve to predict the risk of elevated intraocular pressure at 6 a.m.

**Results:**

Forty-nine (50%) eyes had intraocular pressure measurements at 6 a.m. >21 mmHg, and the median intraocular pressure peak in these eyes at 6 a.m. was 26 mmHg. The best predictors of intraocular pressure measurements >21 mmHg at 6 a.m. were the intraocular pressure measurements at 8 a.m. and concavity. The proposed model achieved a sensitivity of 100% and a specificity of 86%, resulting in an accuracy of 93%.

**Conclusions:**

The machine learning approach was able to predict the risk of intraocular pressure peaks at 6 a.m. with good accuracy. This new approach to the diurnal intraocular pressure curve may become a widely used tool in daily practice and the indica tion of a 24-hour intraocular pressure curve could be rationalized according to risk stratification.

## Abstract (pt)

**Objetivo:**

Utilizar aprendizado de máquina para predizer o risco de picos de pressão intraocular às 6 AM em pacientes com glaucoma primário de ângulo aberto e suspeitos.

**Métodos:**

Esse estudo observacional transversal incluiu 98 olhos de 98 pacientes submetidos à curva de 24 horas de pressão intraocular (incluindo as medidas às 6 AM). A curva diurna de pressão intraocular foi definida como uma série de três medidas da curva de 24 horas de pressão intraocular às 8 AM, às 9 AM e às 11 AM. Duas novas variáveis foram apresentadas: inclinação e concavidade. A inclinação da curva às 8 AM foi calculada como a diferença entre pressão intraocular às 9 AM e 8 AM e reflete a variação da pressão intraocular na primeira hora. A concavidade da curva foi calculada como a diferença entre as inclinações às 9 AM e às 8 AM e pode ser para cima ou para baixo. Uma árvore de classificação foi usada para determinar um algoritmo multivariado a partir das medidas da curva diurna para prever o risco de pressão intraocular elevada às 6 AM.

**Resultados:**

Quarenta e nove (50%) olhos apresentaram pressão intraocular às 6 AM >21 mmHg e a mediana do pico de pressão intraocularPIO foi 26 mmHg. Os melhores preditores de pressão intraocular às 6 AM >21 mmHg foram a pressão intraocular às 8 AM e a concavidade. O modelo proposto apresentou uma sensibilidade de 100% e uma especificidade de 86%, com uma acurácia de 93%.

**Conclusões:**

A abordagem de aprendizado de máquina foi capaz de prever o risco de picos de pressão intraocular às 6 AM com uma boa acurácia. Essa nova abordagem para a curva diurna de pressão intraocular pode se tornar uma ferramenta amplamente utilizada na prática clínica e a indicação da curva de 24 horas de pressão intraocular pode ser racionalizada de acordo com a estratificação de risco.

## INTRODUCTION

Glaucoma is a progressive optic neuropathy characterized by degeneration of retinal ganglion cells and their axons that results in visual field loss and a characteristic appearance of the optic disc^(1)^. Therapeutic strategies in glaucoma aim to reduce the IOP in order to prevent or delay the course of the disease, and once treatment is started follow-up is usually performed through IOP measurements taken during office hours. However, these measurements represent only a small sample of all of the circadian variation of the IOP^(2)^. Lack of a more complete assessment could explain, at least in part, why some patients may experience worsening of glaucoma even though their IOP levels appear to be well controlled.

The importance of carrying out a more complete study of the IOP profile has been demonstrated for dec ades. In 1952, Duke-Elder stated that phasic IOP variations in glaucoma frequently had a large amplitude (quite commonly of 10 or 20 mmHg), occurring at an inconvenient hour^(3)^. Later, Drance reported that almost half of glaucoma patients had IOP peaks at 6 a.m., which were detected through a 24-hour IOP curve^(4)^. The IOP peaks frequently occur upon waking and can be detected at 6 a.m. in darkness and with the patient in a supine position^(5,6)^. Because of the difficulties in taking measurements for the 24-hour IOP curve in clinical practice, the diurnal IOP curve was proposed as an option to evaluate the IOP profile of glaucoma patients. However, as previously mentioned, relying on IOP measurements taken during office hours is not recommended, because they often underestimate IOP peaks^(6-9)^, which may potentially be related to glaucoma progression^(10-13)^.

The purpose of this study was to use a machine learning (ML) approach and the diurnal IOP curve (measured in the morning during office hours) to predict the risk of IOP peaks upon waking at 6 a.m. appearing in a 24-hour IOP curve in patients with primary open-angle glaucoma (POAG) and glaucoma suspects (GS).

## METHODS

This was a cross-sectional study consisting of consecutive POAG patients and GS from the Hospital Sao Geraldo, Federal University of Minas Gerais, Belo Horizonte, Brazil, who underwent 24-hour IOP monitoring. Approval from the institutional review board was obtained for this study, and it was conducted in accordance with the Declaration of Helsinki. Written informed consent was obtained from all participants after the test procedures were explained.

All participants underwent a comprehensive ophthalmologic examination, including visual acuity, slit-lamp biomicroscopy, IOP measurement, gonioscopy, dilated fundoscopy examination using a 78-diopter lens, stereos copic optic disc photography (Canon CR2, Canon USA, Inc., Melville, NY), and central corneal thickness (CCT) measurements using ultrasound pachymetry (DGH 5100, DGH Technology, Inc., Philadelphia, PA). Only subjects with open angles on gonioscopy were included. Standard automated perimetry (SAP) tests were performed using the FASTPAC 24-2 strategy on the Humphrey Field Analyzer II, model 745 (Carl Zeiss Meditec, Inc., Dublin, CA). Reliable visual fields were required to have fixation loss <33% and a false-positive rate <15%. Subjects were excluded if they had other ocular or systemic diseases that could affect the optic nerve or the visual field or if they had undergone glaucoma filtration surgery.

The POAG patients had abnormal visual field test results or a glaucomatous-appearing optic disc [at least one of the following: a cup/disc ratio (C/D) >0.6 in one or both eyes or a C/D asymmetry >0.2] based on stereoscopic optic disc photography assessment. An abnormal visual field was defined as a typical glaucomatous defect in a reliable SAP with a mean deviation (MD) worse than -2 dB and a corrected pattern standard deviation outside of the 95% normal confidence limits. The GS had a history of IOP consistently >21 mmHg and/or a suspicious appearance of the optic nerve [at least one of the following: a (C/D) ratio >0.6 in one or both eyes or a C/D asymmetry >0.2] but normal and reliable SAP results. Each patient was treated at the discretion of the attending ophthalmologist.

### 24-hour IOP curve

During the 24-hour IOP monitoring, the patients were hospitalized and the IOP was measured at 9 a.m., 11 a.m., 6 p.m., 10 p.m., 6 a.m., and 8 a.m. The measu rements were obtained by the same examiner (SC) with the patient in the seated position from 9 a.m. to 10 p.m. using a Goldmann tonometer (Haag-Streit, Köniz, Switzerland) and in the supine position at 6 a.m. and 8 a.m. using a Perkins tonometer (Haag-Streit, Köniz, Switzerland). After the IOP was measured at 6 a.m. in a dark room (IOP6), the patients were asked to leave the bed until 7 a.m. when they were asked to lay down again. After 60 minutes in the same supine conditions, IOP measurements were obtained at 8 a.m. (IOP8). The eye with a higher IOP6 was selected from each patient.

### Diurnal IOP curve

**Figure 1:**
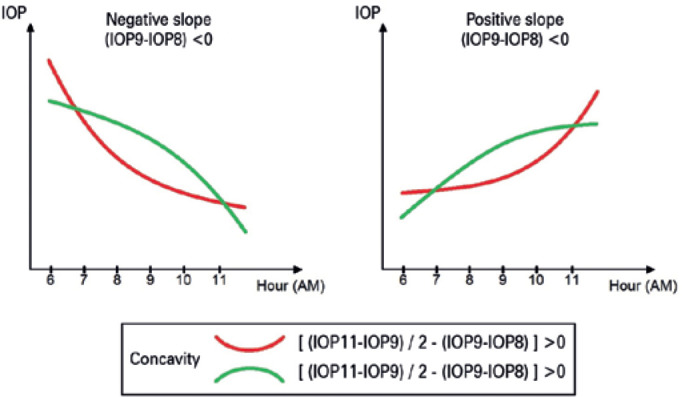
Definitions of slope and concavity of the diurnal IOP curve. Curves with negative slope and positive concavity are expected to show higher IOP6.

The diurnal IOP curve was defined as a series of three IOP measurements taken in the morning to reflect what occurs in daily practice. The diurnal curve included only 24-hour IOP curve measurements at 8 a.m. (IOP8), 9 a.m. (IOP9), and 11 a.m. (IOP11). To analyze the diurnal IOP curve, we introduced two new variables: slope and concavity. These two variables were derived from the mathematical definitions of first and second derivatives. The slope of the diurnal curve at 8 a.m. (S8) was defined as the difference between IOP9 and IOP8 (S8= IOP9 - IOP8) and reflected the IOP change per hour. A negative S8 indicated that the IOP decreased from 8 a.m. to 9 a.m., whereas a positive S8 indicated the opposite. A higher IOP6 is expected in a curve with a negative S8 compared to that in a curve with a positive S8, as shown in figure 1.

The concavity of the curve was defined as the variation of two consecutive measurements of slope. We estimated the slope at 9 a.m. (S9) as the difference between IOP11 and IOP9 divided by 2, as there was a 2-hour interval between the measurements [S9= (IOP11 - IOP9)/2]. The concavity was calculated as the difference between S9 and S8 and it is represented by the following equation:


Concavity=[(IOP11-IOP9)/2-(IOP9-IOP8)]

A positive concavity indicated that the curve was bent upward, whereas a negative concavity indicated the opposite. A higher IOP6 is expected in a curve with positive concavity compared to that in a curve with a negative concavity, as shown in figure 1.

### Statistical analysis

The variables were compared between subgroups using nonparametric tests, and a two-sided p-value ≤5% was considered significant. The Mann-Whitney U test was used to compare continuous variables and Fisher’s exact test with mid-p adjustment was used to compare categorical variables between the two groups. Proportions were compared among more than two groups using Fisher’s exact test. To estimate the confidence intervals (CIs) of percentages, we used exact intervals (Clopper-Pearson). The CIs of medians were calculated using the bootstrap percentile method^(14)^.

A classification and regression tree (CART), a supervised ML method in which the thresholds of the variables are consecutively obtained, was used to determine a multivariate algorithm to predict elevated IOP6 (>21 mmHg) and to classify patients according to their risk of having an elevated IOP6^(15)^. The tree is built stepwise and the variable included in each node is the one that results in the greater reduction of Gini impurity, a measure of statistical dispersion which has a value of zero when all cases in a group have the same measured outcome^(15)^. The tree was obtained requiring a minimum of 5 patients in each subgroup to avoid overfitting. We then post-pruned the tree to minimize the cross-validation error after 10 cross-validations.

To test the robustness of the prediction model, the data were randomly divided into two subsets: a training set containing 60% and a test set containing 40% of the patient data. The model was built and cross-validated using the training set and then tested using the test set. An overfitted model is expected to have prediction accuracies that are different between the two data sets.

All statistical analyses were performed using R softwarR version 3.5.1 (The R Foundation).

## RESULTS

**Figure 2:**
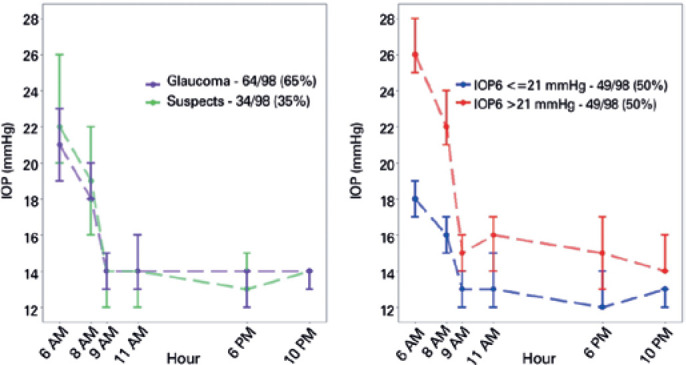
Median and 95% confidence intervals of 24-hour IOP curve measurements according to (1) diagnosis of glaucoma (left) and (2) presence of IOP6 >21 mmHg (right).

Ninety-eight eyes of 98 patients were included in the study. The patient characteristics are shown in table 1 and the median IOP in figure 2, both according to IOP6 values. Fifty-six (57%) patients were female and the median age of all patients was 66 years [interquartile range (IQR): 58-73 years]. Among the 98 patients, 64 were POAG patients and 34 were GS. Of the 64 POAG patients, 25 had mild visual field loss (-6 dB ≤ MD <-2 dB) and 39 had moderate-to-severe visual field loss (MD <-6 dB). Median age was significantly lower in GS when compared with POAG patients (respectively 59 and 68 years; p<0.001). None of the six IOP measurements differed significantly between the POAG patients and the GS, as shown in figure 2.

**Table 1:** Patient characteristics according to intraocular pressure measured at 6 a.m

Baseline characteristics	Total (n=98)	IOP6 ≤21 mmHg (n=49)	IOP6 >21 mmHg (n=49)	P-value
Age (years)	66 [58; 73]	68 [55; 75]	62 [59; 71]	0.442
Female	56/98 (57%)	25/49 (51%)	31/49 (63%)	0.230
Mean deviation (dB)	-4.1 [-11.1; 0.0]	-5.2 [-12.0;-1.4]	-2.4 [-8.6; 0.2]	0.305
Central corneal thickness (µm)	529 [501; 552]	519 [501; 541]	543 [500; 565]	0.164
**IOP (mmHg)**	**Total (n=98)**	**IOP6** ≤**21 mmHg (n=49)**	**IOP6 >21 mmHg (n=49)**	**P-value**
^*^6 a.m.	21 [18; 26]	18 [16; 19]	26 [23; 28]	**<0.001**
^*^8 a.m.	18 [16; 23]	16 [14; 18]	22 [20; 25]	**<0.001**
^*^ 9 a.m.	14 [12; 16]	13 [12; 15]	15 [13; 18]	**0.001**
^*^11 a.m.	14 [12; 16]	13 [12; 16]	16 [13; 17]	**<0.001**
^*^ 6 p.m.	14 [12; 16]	12 [11; 14]	15 [12; 18]	**<0.001**
^*^10 p.m.	14 [12; 16]	13 [11; 14]	14 [13; 17]	**<0.001**
**Diurnal IOP curve characteristics**	**Total (n=98)**	**IOP6** ≤**21 mmHg (n=49)**	**IOP6 >21 mmHg (n=49)**	**P-value**
^*^ S8 (mmHg/h)	-5 [-8; -2]	-3 [-5; -1]	-7 [-10; -5]	**<0.001**
^*^ Concavity (mmHg/h^2^)	5.0 [1.6; 8.4]	3.0 [0.5; 6.0]	7.5 [4.5; 10.5]	**<0.001**

IOP6= intraocular pressure at 6 a.m.; S8= slope at 8 a.m. Continuous variables are expressed as median [IQR] and categorical variables are expressed as absolute numbers (%).

* Significant at α=5%.

Forty-nine (50%) eyes had IOP6 >21 mmHg. Among these patients, the median IOP peak was 22 mmHg (IQR: 18-27 mmHg). Seventy-seven patients (79%) had an IOP peak at 6 a.m. (median 22 mmHg; IQR: 19-27 mmHg), 14 (14%) at 8 a.m. (median 20 mmHg; IQR: 17-24 mmHg), three (3%) at 9 a.m. (median 18 mmHg; IQR: 15-18 mmHg), three (3%) at 11 a.m. (median 15 mmHg; IQR: 13-20 mmHg), zero (0%) at 6 p.m., and one (1%) at 10 p.m. (32 mmHg). All patients who had their maximum IOP measurement during the 24-hour monitoring period at 9 a.m. or at 11 a.m. had IOP6 measurements <21 mmHg. One patient with the maximum measurement during the 24-hour monitoring period at 10 p.m. also had an elevated IOP6 (26 mmHg).

We built a multivariate CART to predict elevated IOP6. The variables included in the model were all of the measurements of the diurnal IOP curve in addition to age, sex, SAP MD, and CCT. The parameters of the model were estimated using only the training set (n=60). After cross-validation, the tree was pruned with two splits, resulting in three subgroups. The best predictors of IOP6 >21 mmHg were IOP8 and diurnal IOP curve concavity.

**Figure 3:**
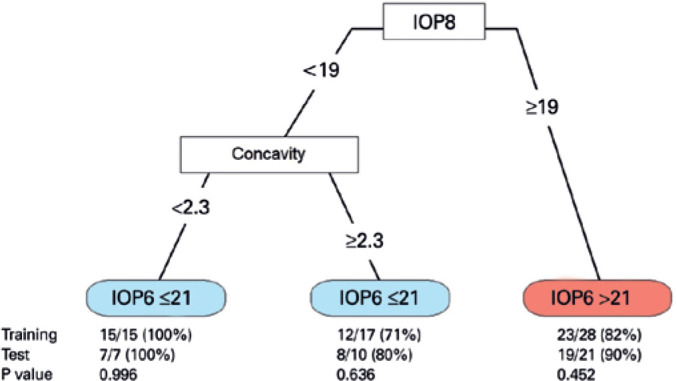
Classification tree to predict elevated IOP6. The best predictors of IOP6 >21 mmHg were IOP8 _≥_19 mmHg and diurnal IOP curve concavity _≥_2.3. The tree was built using only the training set and then applied to the test set in order to test robustness. Proportions (%) of patients with correct prediction in each subgroup are shown for the training and test sets.

Patients were stratified in three subgroups according to the predicted risk of elevated IOP6: a low-risk group (eyes with IOP8 <19 mmHg and concavity <2.3), intermediate-risk group (eyes with IOP8 <19 mmHg and concavity ≥2.3), and high-risk group (eyes with IOP8 ≥19 mmHg). Although 0/15 (0%) from the low-risk group had elevated IOP6, 5/17 (29%) from the intermediate-risk group and 23/28 (82%) from the high-risk group had an elevated IOP6 (Figure 3).

After training, cross-validating, and testing the model, we applied the classification tree to all data in order to summarize our findings. Patients were stratified into the three subgroups according to the predicted risk of elevated IOP6: low-risk (0/22=0%; CI: 0%15%), intermediate-risk (7/27=26%; CI: 11%-46%), and high-risk (42/49=86%; CI: 73%-94%). The three subgroups represented populations with different risks of elevated IOP6, because the proportion of patients with elevated IOP6 within each subgroup was different when they were compared (p<0.001).

**Figure 4:**
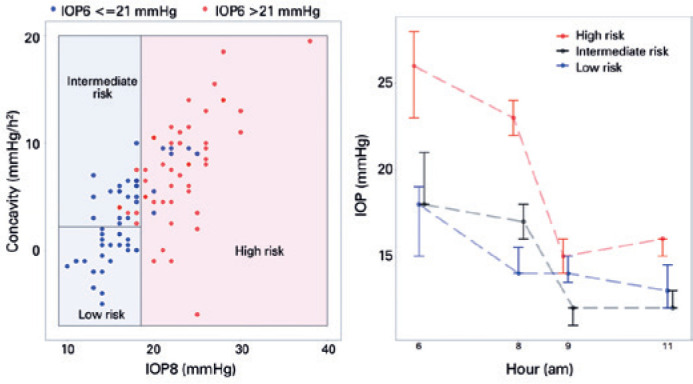
Application of the classification tree algorithm to all data (n=98). Risk zones of elevated IOP6 as a function of IOP8 and diurnal IOP curve concavity (left). Median and 95% confidence intervals of IOP6 and diurnal IOP curve measurements according to risk (points slightly dislocated laterally to avoid overlay) (right).

In figure 4, the risk zones as a function of IOP8 and diurnal IOP curve concavity and the median curves of patients with elevated IOP6 are demonstrated for each subgroup.

The proposed model achieved an accuracy of 97% to predict the risk of morning IOP peak, with 100% sensitivity and 93% specificity, measuring IOP6 only in patients with an intermediate-risk of elevated IOP6 (28% of total). Ninety-one patients (93%) had their maximum IOP measurement during the 24-hour monitoring in the early morning at 6 a.m. or 8 a.m., and all patients with maximum IOP >21 mmHg (53/98=54%) had elevated IOP6 and/or elevated IOP8. If morning IOP peak was defined as either IOP6 or IOP8 >21 mmHg, four patients with IOP6 ≤21 mmHg and IOP8 >21 mmHg, who would be considered false positives in the model to predict elevated IOP6, would become true positives when predicting risk of early morning IOP peak.

## DISCUSSION

Elevated IOP is the most important risk factor for the development and progression of POAG and remains the only known modifiable risk factor^(2,16,17)^. Although average IOP has been consistently established as a risk factor for the development and progression of glaucoma, other IOP parameters, such as 24-hour peaks and fluctuations, have been proposed as potentially related to glaucomatous damage^(10,11,18)^. The 24-hour IOP profile is characterized by several IOP measurements over 24 hours, which makes it possible to reveal peak, fluctuation, and average. All these parameters are interconnected: if a peak occurs, fluctuation and average will automatically increase.

It has been postulated that the occurrence of IOP peaks or fluctuations would be associated with progressive visual field loss in glaucoma^(10-13)^. Sampaolesi et al. demonstrated in 1968 that IOP peaks often occur during the nocturnal sleep period or upon waking, oscillating with circadian rhythm^(5)^, and this was later confirmed^(16,18,19)^. Recently, contact lens sensors (CLS) to continuously monitor IOP were tested^(20)^. Although they were able to characterize the IOP peak timing, CLS do not allow estimation of the IOP value in mmHg^(20)^. Another drawback of these devices is the high rate of adverse effects, such as blurred vision (80%) and conjunctival hyperemia (75%)^(21)^.

In the present study, we proposed a method to predict the risk of elevated IOP6 based on three IOP measurements performed at 8 a.m., 9 a.m., and 11 a.m. This method could possibly overcome the difficulties in obtaining the 24-hour IOP curve in clinical practice and also have great accuracy to predict the risk of IOP peaks in the early morning. We confirmed that IOP peaks are common in the early morning, because 79% of the patients had their maximum IOP measurement during the 24-hour monitoring at 6 a.m. and 14% had it at 8 a.m., in both cases while the patients were in the supine position. Patients with IOP6 >21 mmHg had median IOP values <17 mmHg at both 9 a.m. and 11 a.m. and would be considered as having normal IOP if only these measurements were performed. A comparison of the median values in patients with elevated IOP6 showed that IOP9 was 7 mmHg lower than IOP8 and 11 mmHg lower than IOP6.

We used both supine (IOP8) and sitting (IOP9 and IOP11) IOP measurements to create the diurnal IOP curve to predict the risk of elevated IOP6, because previous studies showed that measurements in the supine position are consistently 2 to 4 mmHg higher than measurements in the sitting position^(16,22,23)^. Two new variables were introduced: slope and concavity. Diurnal IOP curves of patients with elevated IOP6 had lower slope (more negative) and greater concavity than curves of patients with normal IOP6. Six patterns of diurnal IOP curves have been previously described: concave, convex, decreasing, increasing, stable, and sudden break^(24)^. However, these patterns are subjective, limiting their repeatability and applicability in predictive models of elevated IOP upon awakening. We quantitatively described a diurnal IOP curve over a shorter time interval, closer to the 6 a.m. time at which we intended to predict the risk of IOP peak.

As expected, the most important variable to predict risk of elevated IOP6 was IOP8, as it appeared in the first decision node of the multivariate CART. To predict the risk of IOP6 >21 mmHg, a lower threshold for IOP8 (≥19 mmHg) should be used; therefore, we cannot extrapolate IOP8 as an estimate of IOP6 without adjustment, even when IOP8 is measured after one hour in the supine position as suggested by some authors^(16,25)^. The second most important variable to predict elevated IOP6 was the diurnal IOP curve concavity. A concavity <2.3 mmHg/h^^2^^ in patients with IOP8 <19 mmHg virtually excluded the possibility of elevated IOP6, because none of the 22 patients with these characteristics had elevated IOP6. The additional importance of slope and concavity can be demonstrated by the fact that predicting the risk of elevated IOP6 based on only the value of IOP8 (≥19 mmHg) achieved a sensitivity of 86% and specificity of 86%.

It is worth mentioning that ML has been increasingly used in Ophthalmology^(26)^. In glaucoma, deep learning was recently applied to quantify structural damage using optic disc photographs^(27)^. However, some models, such as neural networks, are very difficult to interpret and sometimes are presented as black boxes from which we cannot generate or test meaningful hypotheses^(28)^. The CARTs capture complex nonlinear interactions among variables while keeping model interpretability, allowing easy incorporation of the results into medical practice^(29)^.

The present study has some limitations. As it included a relatively small sample, our results should be viewed as a pilot study to use a ML approach to predict the risk of IOP peaks upon waking at 6 a.m. during a 24-hour IOP curve from a diurnal IOP curve in glaucoma patients. The sample size might have limited the model performance, because we could not allow further partitioning to avoid overfitting. With a larger sample it would be possible to add a suitable validation set, which would increase the performance of the model and provide a better fit. We demonstrated that slope and concavity of the diurnal IOP curve were related to elevated IOP6, but how these variables influence development and progression of glaucoma needs to be further investigated in future studies.

A major limitation of the study was that the patients were treated and therefore IOP-lowering medications may have affected the absolute IOP values, and we were not able to stratify the results by medication. For this reason, the study could not address the effect of hypotensive drugs as a confounding factor during the 24-hour IOP monitoring. Future studies using a ML approach should provide a better understanding of the relation of the diurnal IOP curve to the 24-hour IOP behavior and how each medication affects this circadian rhythm. Finally, because the patients were included from the same referral center and the distribution of IOP may vary across different populations, the results cannot be extrapolated before being validated in other settings and populations.

In conclusion, a ML approach was used to stratify the eyes of patients according to the risk of having an early morning IOP peak, rationalizing the indication of a 24-hour IOP curve. The proposed methodology with two new variables (diurnal IOP slope and concavity) had an accuracy of >90% to predict the risk of IOP peaks in the early morning. This new approach to the diurnal IOP curve may become a widely used tool in daily practice, as it avoids the need for hospitalization.
